# Potential health gains and health losses in eleven EU countries attainable through feasible prevalences of the life-style related risk factors alcohol, BMI, and smoking: a quantitative health impact assessment

**DOI:** 10.1186/s12889-016-3299-z

**Published:** 2016-08-05

**Authors:** Stefan K. Lhachimi, Wilma J. Nusselder, Henriette A. Smit, Paolo Baili, Kathleen Bennett, Esteve Fernández, Margarete C. Kulik, Tim Lobstein, Joceline Pomerleau, Hendriek C. Boshuizen, Johan P. Mackenbach

**Affiliations:** 1 Department of Public Health, Erasmus MC, University Medical Center Rotterdam, Rotterdam, The Netherlands; 2 Research Group for Evidence Based Public Health, Institute for Public Health and Nursing, University Bremen & Leibniz Institute for Epidemiology and Prevention Research, Bremen, Germany; 3 Department of Statistics and Mathematical Modeling, Expertise Centre for Methodology and Information Services, National Institute for Public Health and the Environment (RIVM), Bilthoven, The Netherlands; 4 Department of Public Health, Heinrich Heine University, Duesseldorf, Germany; 5 Julius Center for Health Sciences and Primary Care, University Medical Centre Utrecht, Utrecht, The Netherlands; 6 Center for Prevention and Health Services Research (PZO), National Institute of Public Health and the Environment (RIVM), Bilthoven, The Netherlands; 7 Descriptive Studies and Health Planning Unit, Fondazione IRCCS “Istituto Nazionale Tumori”, Milan, Italy; 8 Department of Pharmacology & Therapeutics, Trinity Centre for health sciences, St James’s Hospital, Dublin, Ireland; 9 Tobacco Control Unit, Institut Català d’Oncologia-IDIBELL, L’Hospitalet de Llobregat Barcelona, Barcelona, Spain; 10 Department of Clinical Sciences, School of Medicine, Campus of Bellvitge, Universitat de Barcelona, Barcelona, Spain; 11 IASO -the International Association for the Study of Obesity, IOTF -the International Obesity TaskForce, London, UK; 12 European Centre on Health of Societies in Transition, London School of Hygiene and Tropical Medicine, London, UK; 13 Division of Human Nutrition, Wageningen University, Wageningen, Netherlands

**Keywords:** Alcohol, BMI, Smoking, Life-style related risk-factors, Health impact assessment, Modeling

## Abstract

**Background:**

Influencing the life-style risk-factors alcohol, body mass index (BMI), and smoking is an European Union (EU) wide objective of public health policy. The population-level health effects of these risk-factors depend on population specific characteristics and are difficult to quantify without dynamic population health models.

**Methods:**

For eleven countries—approx. 80 % of the EU-27 population—we used evidence from the publicly available DYNAMO-HIA data-set. For each country the age- and sex-specific risk-factor prevalence and the incidence, prevalence, and excess mortality of nine chronic diseases are utilized; including the corresponding relative risks linking risk-factor exposure causally to disease incidence and all-cause mortality.

Applying the DYNAMO-HIA tool, we dynamically project the country-wise potential health gains and losses using feasible, i.e. observed elsewhere, risk-factor prevalence rates as benchmarks. The effects of the “worst practice”, “best practice”, and the currently observed risk-factor prevalence on population health are quantified and expected changes in life expectancy, morbidity-free life years, disease cases, and cumulative mortality are reported.

**Results:**

Applying the best practice smoking prevalence yields the largest gains in life expectancy with 0.4 years for males and 0.3 year for females (approx. 332,950 and 274,200 deaths postponed, respectively) while the worst practice smoking prevalence also leads to the largest losses with 0.7 years for males and 0.9 year for females (approx. 609,400 and 710,550 lives lost, respectively).

Comparing morbidity-free life years, the best practice smoking prevalence shows the highest gains for males with 0.4 years (342,800 less disease cases), whereas for females the best practice BMI prevalence yields the largest gains with 0.7 years (1,075,200 less disease cases).

**Conclusion:**

Smoking is still the risk-factor with the largest potential health gains. BMI, however, has comparatively large effects on morbidity. Future research should aim to improve knowledge of how policies can influence and shape individual and aggregated life-style-related risk-factor behavior.

**Electronic supplementary material:**

The online version of this article (doi:10.1186/s12889-016-3299-z) contains supplementary material, which is available to authorized users.

## Background

Life-style related risk factors are major determinants of morbidity and mortality within the EU. Chief among them alcohol [1], overweight [2], and smoking [3]. Inducing citizens to adopt a healthier life-style is currently a major goal of European health policy in order to reduce morbidity and premature mortality from chronic diseases such as cardiovascular diseases, cancer, diabetes, and chronic respiratory disease [4]. Nevertheless, WHO’s European region still has the highest levels of smoking and alcohol consumption in the world, and ranks second in rates of overweight and obesity [5]. Despite some evidence on long-term convergence of exposure to lifestyle-related risk factors among European countries [6], there are still salient differences between countries. Hence, the comparative impact and importance of a risk factor will differ between countries as risk factor prevalences differ between countries. Furthermore, the very same risk factor prevalence might yield different health effects, depending on a number of country specific factors. For example, the current incidence, prevalence, or mortality levels of related diseases, of competing but not directly related diseases, or the population structure itself can differ significantly, modifying the impacts of risk factors on morbidity and mortality. Hence, identifying the contribution of a modifiable risk factor and comparing it with other risk factors in terms of potential health benefits, must account for the specific context in each country. We developed a now publicly available software tool DYNAMO-HIA (DYnamic MOdeling for Health Impact Assessment), designed for projecting and quantifying the effects of risk factor exposure on real-life populations. In this paper, DYNAMO-HIA is used to quantify potential health gains and health losses caused by a change in either of the three risk factors: alcohol consumption, BMI (body mass index), and smoking in eleven EU countries (covering approx. 80 % of the EU population). This gives country-by-country and virtually EU-wide insight for each of these three risk factors which health gains may be achieved and health losses might occur and, hence, which risk factors are most promising to target and most hazardous to neglect. Previous health impact assessments focused either on specific diseases clusters (e.g. cancers [7] or cardiovascular diseases [8]), used non-dynamic projection methods [9] or selected age groups [10], focused on single risk factors [11] or countries [12], and/or used only theoretically feasible minimum risk factors exposure levels for intervention scenarios [13]. This is—to the best of our knowledge—the first dynamic health impact assessment [14] comparing the effects of these three risk factors not only for life expectancy but also jointly considering chronic obstructive pulmonary disease (COPD), heart diseases, diabetes, and major cancer types across several countries.

## Methods

Quantification of potential health gains and losses due to changes in risk factor exposure requires defining sensible counterfactual scenarios as benchmarks. Those benchmark scenarios are compared to a reference scenario, which for each country is based on the currently observed risk factor prevalence (also known as “business-as-usual” scenario). Hence, for each risk factor/country combination three scenarios are dynamically projected into the future, i.e. the “business-as-usual” scenario and a “upper” exposure level and a “lower” exposure level as the counterfactual scenarios that quantify potential health gains and health losses.

### Data sources

The DYNAMO-HIA project compiled a publicly available EU-wide data set of which eleven countries have complete disease and risk factor information [15–19] (See www.dynamo-hia.eu for full documentation). For each country, we use age- and sex-specific data on the population (size, projected birth numbers, and total mortality rate)—as well as prevalences for BMI, alcohol consumption, and smoking behavior (exception: no smoking data for Poland). Furthermore, nine major chronic diseases are included: ischemic heart diseases (IHD), diabetes, COPD, stroke, and lung-, breast-, colorectal, oral, and esophageal-cancer. Each disease is characterized by incidence, prevalence, and excess mortality. For stroke and IHD, excess mortality is modeled via two elements: (a) age- and sex-dependent increases in mortality when having those diseases and (b) acute increased mortality when contracting the disease to reflect that for those diseases mortality is higher at time of incidence. Where appropriate, missing data have been back-calculated using DISMOD II [20], applying the mathematical relationship between incidence, prevalence, and case-fatality-rate for chronic diseases within a given population. In addition, DISMOD II was used to ensure smooth and internally consistent data. Lastly, we use data on age- and sex-specific relative risk causally connecting the risk factors to disease incidence and the relative risks of IHD- and stroke-incidence when having diabetes.

### Counterfactual benchmarks

Quantifying potential health gains or losses requires the definition of counterfactual risk factor prevalences as a benchmark. We opted for *feasible* prevalence rates, i.e. empirically observed prevalence rates across countries, to increase policy relevance of the analysis. Previous studies often use theoretically motivated feasible minimum risk factors exposure levels as counterfactuals—such as eradication of overweight or smoking—that are unrealistic to achieve in the near future by government policy. For example, a policy can only turn a current smoker into a former smoker and not into a never smoker Hence, (past) smoking behavior will be contributing to the overall disease burden in a population at least for some time.

The approach we used to construct feasible risk factor prevalences is related to the calculation of best practice life expectancies within the field of demography [21]. For that, we compared each age and sex group across the eleven countries. For each particular age/sex combination we selected as the counterfactual prevalence the prevalence observed in the “best” or “worst” practice country, by comparing the highest proportion in the most desirable risk factor category for “best” practice and the highest proportion in the least desirable risk factor category for “worst” practice in this particular age/sex combination. This approach can be interpreted as a synthetic population that at every age exhibits the “best” (“worst”) health behavior among all feasible, i.e. empirically observed, health behaviors for this particular age and sex. In our analysis the overweight and obesity category in adults were based on those defined by WHO [22]. Hence, for BMI the most desirable risk factor category is a BMI of 25 or less (as this has a relative risk of 1 for all-cause mortality) and the least desirable risk factor category is a BMI >30 as this is a condition that should be avoided. For smoking, the most desirable categories are never- and former-smokers (as both are behaviors that ought to be retained) and the least desirable category is current-smokers (occasional and daily smokers) when considering the relative risk for mortality [3]. Clearly, being a never-smoker is more desirable than being a former smoke. However, the most desirable state a current smoker can be induced to occupy by policy is “former smoker”. For alcohol the most desirable risk factor categories are a consumption of pure alcohol of <20 g/day (gram per day) and the least desirable categories are a consumption of more than 40 g/day when accounting for the relative risk of overall mortality [23]. For the best practice scenario we keep the share of abstainers constant as it would be unethical if a policy aims to induce abstainers to become (light) drinkers [24]. (See Table 1 for an example calculation and Additionla file 1 for a graphical depiction.)

**Table 1:** Example of counterfactual construction

For 50 year old males, for example, the UK (United Kingdom) has the highest proportion of individuals in the undesirable alcohol consumption categories among the eleven countries (some 31 % consume more than 40 g/day). Hence, the observed alcohol consumption prevalence for this particular age/sex group is used for constructing the worst practice counterfactual scenario for the age- and sex group of 50 year old males. For 33 year old females, for example, Denmark has the highest proportion of citizens with a desirable BMI (some 80 % have a BMI < 25). Hence, the observed BMI prevalence for this age/sex group is used for the corresponding age/sex group in the best practice counterfactual. Often one or two countries provide most observations for a given risk factor/sex combination. For example, for the best practice BMI prevalence for females all values above age 30 are taken from Denmark as those have the highest proportion of females with a BMI < 25. In the case of alcohol, for example, the worst practice counterfactual for males is identical to the observed prevalence of the UK.	

Using this approach, we acknowledge that from a policy perspective the risk factor behavior of an individual is the smallest, amenable unit of analysis: retaining desirable behavior and reducing non-desirable behavior. This criterion values each individual and population equally, i.e. age, age structure, and population size do not have an influence. Other, health-outcome-focused approaches to select a desirable (or undesirable) prevalence among several observed prevalences usually bias the selection towards the used outcome measure. For example, attributable deaths as a decision criterion would be largely determined by exposure among the old as those contribute to the majority of deaths in a population; life years lost would shift the focus towards risk factor exposure among the young.

### Dynamic modeling

Within the DYNAMO-HIA consortium (www.dynamo-hia.eu), we designed and implemented a publicly available software tool that quantifies the effect of changing risk factor exposure on population health [25, 26]. DYNAMO-HIA was specifically developed to quantify the effects of difference between one or more risk factor exposures—such as smoking [27], alcohol consumption [25], obesity [28], salt intake [29], physical activity [30], or second hand smoking [31]—on different health outcomes, such as prevalence of specific diseases, overall mortality and summary measures of population health. As a population health model, the DYNAMO-HIA tool allows to assess effects of risk factor changes on a real-life population, hence, accounting for the country-specific population composition and the incidence/prevalence/mortality-profile [32] of relevant diseases. Dynamic modeling assesses the development of population health in the short- and in the long-run while accounting for population aging—a process that is rapidly taking place in most EU countries.

At the core, DYNAMO-HIA is Markov-type based model simulating a real-life population (birth and death but no migration). It projects the future risk factor exposure and, consequently, the annual disease incidence and resulting prevalence of chronic diseases and mortality over time, accounting for competing risks (see the Additional file 1 for a description of the model core, an article with a general explanation of the methodology has been published here [33], and a an article explaining the algorithms used has been published here [25]). For the specification of a scenario, DYNAMO-HIA requires information on the starting risk factor prevalence and how risk factor exposure will develop in the future. For every subsequent year, DYNAMO-HIA applies to each age and sex group a probability determining the proportion of individuals that will stay in this risk factor group or will move to another risk factor group (e.g. how many will keep their normal weight or will become overweight or obese in the next year), and hence change the risk of disease incidence and mortality in addition to the effect of aging. The specification of these (future) transition probabilities influences greatly the development of the risk factor prevalence over time. Future individual risk factor behavior is always embedded with uncertainty and, hence, debatable. One approach [34]—that DYNAMO-HIA provides—is to use net transition probabilities, i.e. transition rates that keep the age-specific risk factor prevalence constant, not taking into account any future cohort effects. This is done for all three scenarios (“best”, “worst”, “business-as-usual”) in each country/risk factor combination leading to 96 distinctive scenarios (for Poland smoking data is not available).

### Outcome measures

DYNAMO-HIA provides a wide range of outcome measures of which only three are reported here. (i) Absolute Numbers: As migration is assumed to be zero and projected births do not differ between scenarios, the difference in population size can be interpreted as the cumulative number of deaths postponed or lives lost during the ten year projection period. However, absolute numbers have to be interpreted with caution. Some of the results can be solely driven by one or two large countries that are more strongly affected by changing a risk factor than many smaller countries, for example, through a different age-structure or unequal exposure between parts of the population.

(ii) Life expectancy (LE) as a well-established and robust summary measure of the current mortality regime of a population that is uninfluenced by its population size and age structure.

(iii) Morbidity-free life expectancy (MFLE), i.e. years without chronic morbidity is calculated using the Sullivan method [35], in ten years from now. Like period life expectancy, MFLE is independent of the age structure of a population while combining current mortality and morbidity conditions in a single summary measure. It measures the average numbers of years a newborn can expect to live in good health, i.e. without any of the diseases included in our projection, under current conditions.

## Results

This section provides an overview of the projection results. Detailed tables are available in the Additional file 1.

### Absolute numbers

Tables 2 and 3 give an overview of changes in absolute prevalences and deaths postponed/lives lost when adopting the best and worst practice counterfactual, respectively, for all countries combined (without Poland to allow comparison across risk factors).

**Table 2:** Comparison of best practice risk-factor exposure and reference scenario across all countries^a.^ Difference in disease cases by disease and by persons with at least one disease in projection year ten and cumulative number of deaths postponed as calculated by the difference in individuals alive between the scenarios by sex and risk-factor

	Males			Females		
	Alcohol	BMI	Smoking	Alcohol	BMI	Smoking
Breast cancer	n/a	n/a	n/a	−44,650	−46,250	18,100
Colorectal cancer	−40,050	−20,200	10,250	−8,150	−8,750	6,050
COPD	2,700	2,400	−154,600	3,900	3,850	−291,550
Diabetes	−232,600	−481,800	46,750	82,900	−924,550	34,150
Esophageal cancer	−8,350	50	−4,650	−1,550	50	−3,350
IHD	−89,400	−244,900	−189,600	113,500	−304,550	−147,750
Lung cancer	500	16,900	−52,450	350	8,800	−34,750
Oral cancer	−49,000	12,150	−32,900	−10,300	5,200	−8,650
Stroke	−126,950	−86,750	−148,750	103,900	−147,500	−124,000
At least one of the above diseases	−399,800	−595,650	−342,800	157,650	−1,075,200	−349,300
Deaths postponed^b^	93,750	57,500	332,950	134,050	129,750	274,200

^a^Without data for Poland for to allow comparison

^b^Calculated as the difference in population size between the respective scenario if migration is zero and number of birth constant

**Table 3:** Comparison of worst practice risk-factor exposure and reference scenario across all countries^a.^ Difference in disease cases by disease and by persons with at least one disease in projection year ten and cumulative number of lives lost as calculated by the difference in individuals alive between the scenarios by sex and risk-factor

	Males			Females		
	Alcohol	BMI	Smoking	Alcohol	BMI	Smoking
Breast cancer	n/a	n/a	n/a	83,450	27,250	−47,750
Colorectal cancer	42,150	19,450	−19,500	18,650	3,300	−15,650
COPD	−2,200	−2,850	250,600	−1,500	−3,400	824,450
Diabetes	92,100	619,850	−98,100	14,600	724,800	−103,550
Esophageal cancer	8,500	−100	9,900	1,200	−50	9,800
IHD	65,000	183,750	215,100	3,800	176,450	540,600
Lung cancer	−300	−13,750	111,800	−50	−6,100	119,450
Oral cancer	60,950	−12,700	85,350	15,300	−4,050	357,50
Stroke	99,600	79,450	307,800	117,000	103,750	443,950
At least one of the above diseases	274,000	681,850	537,500	188,300	771,000	1,190,300
Lives lost^b^	110,250	68,500	609,400	69,550	138,950	710,550

^a^Without data for Poland to allow comparison

^b^Calculated as the difference in population size between the respective scenario if migration is zero and number of birth

Adopting the best practice smoking scenario in each country would postpone over half a million deaths as compared to the reference scenario. In terms of disease prevalence, BMI has the greatest potential in reducing the absolute case load, increasing the number of healthy people by over 1.6 million. Adopting the best practice alcohol prevalence would translate for females into some 130,000 deaths postponed but would also increase the disease load substantially. This increased disease prevalence is partly caused by the fact that more people are alive and, hence, susceptible to causally unrelated diseases, e.g. COPD cases in the alcohol and BMI scenarios, but also by the beneficial effect of light alcohol consumption for parts of the population.

Deteriorating to the worst practice for smoking would lead to the largest number of premature deaths with some 1.3 million with almost equal effects for both genders. However, the number of diseased among females would rise to 1.2 million as compared to the reference scenario. In men, the number of diseased males exceeds that of the reference scenario with 540,000 cases. The reason for this seemingly gender disparity between of additional number of deaths and disease cases when deteriorating the smoking prevalence to the worst practice lies in a complex interplay of several factors. One is that men have a 20 % higher risk for dying from smoking than females. Another factor is that the worst practice counterfactuals affect the genders unequally. For large countries—Germany, Italy, France, Spain—the number of females smokers in the worst practice scenario increases substantially among the middle aged where diseases are contracted but general mortality is lower; hence, the effect of an increased relative risk (RR) through smoking status on the absolute number of death is lower than at higher ages.

For BMI, the worst practice exposure would lead to a similar increase of number of diseased (771,000 for females vs. 681,000 for males), but females would suffer more than twice as many premature deaths than males (140,000 vs. 70,000). This is partly explained by some countries, in particular Germany and the UK, where in the reference scenario the proportion of obese women among the older females, where in absolute numbers the majority of attributable deaths occur, is much lower than in the worst practice scenario.

An adoption of the worst practice alcohol prevalence would lead to an increase in the number of disease cases and lives lost for both genders, whereas males would be stronger affected than females. The comparatively lower increase in IHD cases for females compared to males is partly explained by the fact that only a daily consumption of more than 60 g/day has a RR larger than one and the proportion of females in this highest drinking category increases only barely in the worst practice scenario, while it increases considerably more in males.

### Life expectancy

**Fig. 1:**
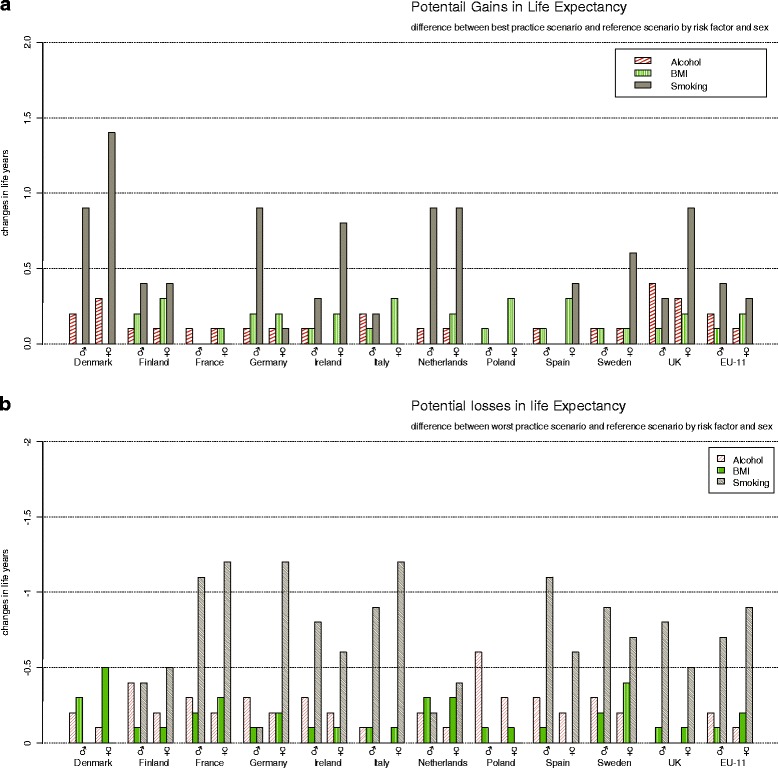
Life expectancy*. **a** Potential gains in life expectancy. **b** Potential losses in life expectancy. Potential gains in life expectancy (Panel **a**) and potential losses in life expectancy (Panel **b**) as measured by the differences in period life expectancy after ten years for each country and all eleven countries (EU-11) combined by risk-factor and sex compared with the reference scenario. *No smoking data for Poland

Figure 1 shows the differences in period life expectancy when adopting the best and worst practice counterfactual, panel (a) and (b) respectively, after ten years. Smoking is the risk factor that has by far the biggest potential to achieve total gains in LE with 0.4 years (y) for males and 0.3y for females when all countries would adopt the best practice prevalence and, similarly, the largest losses with 0.7y for males and 0.9y for females when across all countries the worst practice smoking prevalence would be adopted. The effects of changes in alcohol and BMI prevalence differ by sex. At the aggregated level, male LE is more affected by alcohol than female LE. The potential increase and the potential loss in LE have a magnitude of 0.2y for males as compared to 0.1y for females. This magnitude, however, is reversed for BMI where the potential increase and the potential loss in life expectancy have a magnitude of 0.2y for females and only 0.1y for males.

The comparatively large effect of smoking is explained through the large relative risks of smoking on several diseases—including lung cancer, COPD, IHD, and stroke—and a large variation in smoking exposure patterns across countries. The differences between worst practice and best practice smoking prevalences for both genders are considerable. Denmark, for example, has among the 60 year olds a more than three times higher female smoking prevalence than Italy and a more than two times higher male smoking prevalence than France. For alcohol the range is smaller and differs by gender. In most countries females drink in general less often hazardous quantities. However, the worst practice counterfactual–almost uniquely based on the UK prevalence—is in comparison unusually high. For males, the differences in the risk factor prevalence between scenarios are in most countries generally less large in relative terms but still sizeable in absolute terms, in particular for younger ages, e.g. below 45. For obesity, the range between the worst practice counterfactual and the prevalence of obesity in the reference scenario for most countries is in particular high for ages above 75.

### Morbidity-free life expectancy

**Fig. 2:**
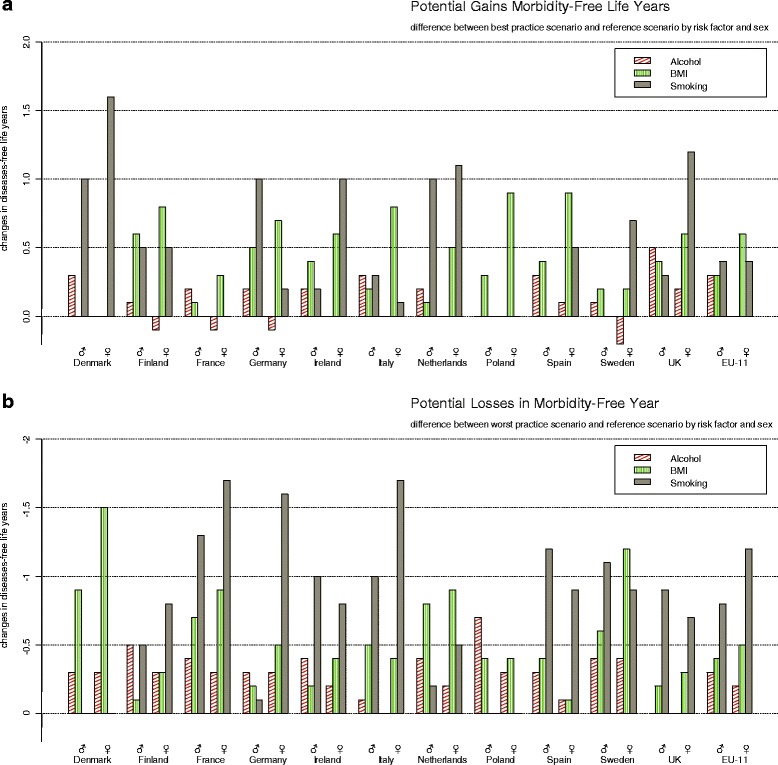
Morbidity-free life years*. **a** Potential gains in morbidity-free life years. **b** Potential losses in morbidity-free life years. Potential gains in morbidity-free life years (Panel **a**) and potential losses in morbidity-free life years (Panel **b**) as measured by the differences in disease-free life years after ten years for each country and all eleven countries (EU-11) combined by risk-factor and sex compared with the reference scenario. *No smoking data for Poland

Figure 2 shows the projected differences in morbidity-free life expectancy (MFLE).

For males, the gains and losses in morbidity-free years differed less between risk factors as compared to those in LE, reflecting similar effects of smoking on MFLE than on LE, but larger effects of alcohol and BMI on MFLE than on LE. Hence, adopting the best practice smoking prevalence rate would lead to only to slightly larger gains in disease-free years (0.4y) than alcohol and BMI (both 0.3y). Deteriorating to the worst practice prevalences for smoking would lead to a loss of 0.8 morbidity-free life years as compared to 0.3y for alcohol and 0.4y for BMI.

For females, the higher relative importance of obesity for MFLE gains and losses than for LE gains and losses is most notable. Pursuing the best practice prevalence for obesity promises gains of 0.7y without morbidity compared to 0.4y for smoking. Furthermore, adopting the best practice prevalence for alcohol would not lead to any gain in years free of morbidity whereas the worst practice would lead to a loss of 0.2y. When regressing to the worst practice, smoking would have the largest effect with a loss of 1.2 morbidity-free years followed by obesity with 0.5 morbidity-free years. This narrowing of the differences is partly explained through the different effects of these risk factors on health. Smoking causes mostly illnesses with substantially higher excess mortality and hence shorter disease duration whereas obesity increases the number of chronic diseases with comparably lower excess mortality and thus longer duration. For alcohol, it is important to note that moderate consumption has beneficial effects at higher age, reducing the incidence of a number of chronic diseases.

## Discussion

For eleven EU countries, we benchmarked potential health gains and losses by comparing the current risk factor prevalence with a best practice and a worst practice scenario, respectively. The comparison was done using a dynamic population health model, DYNAMO-HIA, projecting for each country the real-life population with the corresponding incidence/prevalence/mortality profile of the nine included diseases for ten years into the future, yielding a variety of health outcome measures.

Smoking is still the most salient risk factor for both genders in the EU. The potential gains, when adopting the best practice, and the possible losses, when deteriorating towards the worst practice, are immense in terms of both mortality and morbidity (approx. 1.3 million lives lost over a ten year period). When comparing alcohol with BMI, alcohol as a factor for mortality is more influential for males, i.e. more male deaths could be postponed when adopting the best practice alcohol prevalence and more male lives would be lost when deteriorating towards the worst practice alcohol prevalence as compared to the respective BMI scenarios. For females however, the difference between the best practice BMI prevalence and the worst practice BMI prevalence has a larger effect on life expectancy across countries as compared to the respective alcohol scenarios. For both genders, BMI as a risk factor in terms of morbidity, however, is weightier than alcohol in absolute prevalence cases across countries and morbidity-free life years. Hence, for males in some countries (e.g. Germany) targeting BMI is the risk factor yielding larger gains in increases in life expectancy and morbidity-free life years than targeting alcohol.

The results of this modelling study confirm the importance of life-style related risk factors on population health for affluent countries like those included in our analysis. By focusing on feasible risk factor prevalences, our results are on average smaller than in studies focusing on the total burden of disease attributable to these risk factors or on all avoidable diseases [36, 37]. Nevertheless, in terms of magnitude of the expected changes in life expectancy, our results are in line with a study dynamically modeling changes in life expectancy for the population of the United States caused by a continuation of trends in smoking and obesity [38, 39].

Future research should aim to improve our knowledge of how policies can influence and shape individual and aggregated life-style related risk factor behavior. Our analysis, however, cannot be used to directly inform policy action, its findings rather show where to focus efforts. It would be worth to scrutinize those countries that for some or all their population have best (or worst) practice behavior to identify what are the differences that could explain differences in modifiable factors. These causes, such as tax or market regimes, could aid future policy making tremendously [40–42].

### Limitations

The main outcome measure of our study are mortality and morbidity. Expressing the changes in mortality and morbidity as life expectancies and morbidity-free life expectancies, respectively, allows comparisons across countries as life expectancy is independent of the underlying age structure. Quantifying changes morbidity as disease prevalence is affected by population size and age structure. However, the morbidity measures used do not distinguish the severity of the different diseases and morbidity-free life expectancy only accounts for the nine diseases projected. Although all major diseases that are causally related to at least one of the three risk factors are included other diseases exist that affect this measure.

For smoking a complication lies in the handling of former-smokers as those is not a truly desirable risk factor state but current-smokers cannot go back to become never-smokers. Although from a health perspective only never-smoking is a seemingly desirable risk factor state, a policy perspective must acknowledge that for many individuals becoming or staying former-smokers must be the policy goal.

Similarly, light drinking of alcohol decreases the risk of mortality as well as incidence of stroke and IHD as compared to abstainers. For some countries—namely Sweden, Germany, and Finland—adopting the best practice scenario, while increasing the overall proportion of females with a desirable drinking behavior, leads to a decrease of the number of light drinkers while increasing the numbers of abstainers. A significant limitation in the results for alcohol, however, lies in the unspecified effect that changes in overall alcohol consumption have on those that do not change their consumption, e.g. through violence or accidents committed by intoxicated individuals. The relative risks only account for the effect on those who drink. Thus, the causal pathway of alcohol on health is as much a biological as a social one, making an universally optimal consumption level and, hence, best practice scenario difficult to define. Moreover, all three risk-factors have effects beyond the health measures reported in this analysis, such as impacting long-term labor market outcomes [43–45].

DYNAMO-HIA compares the effects of intervention/policies, i.e. quantification of a reference scenario and one or more intervention scenarios with a modified risk factor exposure. The goal is not to project future population health as such. For projecting future population health, accurate information on incidence, prevalence, and excess mortality data of the diseases included in the model are needed in order to predict such future trends, while those data in reality are embedded with uncertainty. This is partly because of the presence of past trends which are not exactly known. For the DYNAMO-HIA database it was decided to include trend-free incidence/prevalence/mortality-data partly estimated using DISMOD II software. Such trend-free data are used as a neutral option, because of the lack of reliable information on trends. In view of the intended use of DYNAMO-HIA—that is, comparing scenarios—this choice is not very significant as the same disease data are used both in the intervention and reference scenario(s). Therefore we do not expect that this unavoidable compromise has an important effect on the outcomes of our study.

We focus on a short projection span of ten years to increase policy relevance of the application. Furthermore, longer projection periods may show larger magnitudes in terms of health outcomes, but those outcomes would become increasingly less certain as they would be more influenced by cohort effects. Moreover, we assume that the change in the risk-factor prevalence in the intervention scenarios takes place immediately, e.g. without any adjustment period. A real life policy change, however, takes time to come to full effect and potentially also induces dynamic response in health behavior.

## Conclusion

Employing feasible counterfactuals as benchmarks for potential health gains and losses across eleven EU countries indicates that smoking is still the most salient risk factors in terms of morbidity and mortality in both men and women, with a notable exception: female morbidity could be reduced most when focusing on BMI as a risk-factor. For males, alcohol is more important than BMI in terms of mortality and vice versa for females. In terms of morbidity BMI is more important or at least equally important as alcohol for both genders.

## Additional file

Additional file 1: Overview eAppendix. (DOCX 590 kb)
