# Supplementary material for: Potential health gains and health losses in eleven EU countries attainable through feasible prevalences of the life-style related risk factors alcohol, BMI, and smoking: a quantitative health impact assessment
Source: BMC Public Health. 2016 Aug 5;16:734. doi: 10.1186/s12889-016-3299-z (PMC4975898; doi:10.1186/s12889-016-3299-z)
Supplement: Additional file 1: — Overview eAppendix. (DOCX 590 kb) [file 12889_2016_3299_MOESM1_ESM.docx]

**Overview eAppendix**

[eFigure 1 Risk factor prevalences for Alcohol](#_Toc337392117)

[eFigure 2: Risk factor prevalences for BMI](#_Toc337392118)

[eFigure 3: Risk factor prevalences for smoking](#_Toc337392119)

[eTable 1 Overview of deaths postponed/lives lost as compared to the business-as-usual scenario (by country)](#_Toc312664668)

[eTable 2: Country wise difference in cases of having any disease](#_Toc312664669)

[eTable 3 Country wise overview of disease prevalence for included diseases.](#_Toc312664670)

[eTable 4: Overview of relative risks from alcohol to diseases and total mortality (below the age of 15 all relative risks are set 1)](#_Toc312664671)

[eTable 5: Overview of relative risks from smoking to diseases and total mortality used in the example applications (below the age of 35 all relative risks are set to 1)](#_Toc312664672)

[eTable 6: Table of estimated relative risks of disease linked to overweight and obesity](#_Toc312664673)

[eTable 7: Overview of relative risks from diabetes to IHD and stroke used in the example applications](#_Toc312664674)

eFigure 1 Risk factor prevalences for Alcohol

eFigure 2: Risk factor prevalences for BMI

eFigure 3: Risk factor prevalences for smoking

eTable 1 Overview of deaths postponed/lives lost as compared to the business-as-usual scenario (by country)

| a) Death postponed in best practice | | | | | | | |  |  | b) Lives lost in worst practice | | | | | | | | | |
| --- | --- | --- | --- | --- | --- | --- | --- | --- | --- | --- | --- | --- | --- | --- | --- | --- | --- | --- | --- |
|  |  | **Males** |  |  |  | **Females** |  |  |  |  | **Males** |  | |  |  | | **Females** | |  |
|  | **Alcohol** | **BMI** | **Smoking** |  | **Alcohol** | **BMI** | **Smoking** |  |  | **Alcohol** | **BMI** | **Smoking** | |  | **Alcohol** | **BMI** | | | **Smoking** |
| Denmark | 1,700 | 0 | 12,900 |  | 4,900 | 0 | 16,600 |  | Denmark | -1,800 | -2,800 | -300 |  | | 500 | | | -6,200 | -100 |
| Finland | 900 | 2,300 | 5,900 |  | 1,300 | 3,000 | 5,600 |  | Finland | -3,600 | -400 | -5,400 |  | | -2,500 | | | -2,300 | -6,600 |
| France | 6,500 | 1,900 | 7,000 |  | 26,100 | 7,000 | -600 |  | France | -30,900 | -15,900 | -163,200 |  | | -10,800 | | | -29,700 | -147,400 |
| Germany | 14,600 | 22,700 | 186,700 |  | 45,300 | 36,200 | 38,500 |  | Germany | -34,700 | -8,400 | -15,000 |  | | -23,100 | | | -36,900 | -250,400 |
| Ireland | 600 | 700 | 2,600 |  | 400 | 1,100 | 5,300 |  | Ireland | -1,700 | -400 | -5,900 |  | | -1,500 | | | -1,100 | -4,500 |
| Italy | 18,500 | 7,200 | 39,300 |  | -3,300 | 31,100 | 7,300 |  | Italy | -8,200 | -15,300 | -151,900 |  | | -2,100 | | | -22,100 | -174,300 |
| NL | 1,800 | 200 | 35,600 |  | 7,500 | 5,500 | 30,600 |  | NL | -6,400 | -7,700 | -7,400 |  | | -4,200 | | | -11,500 | -13,800 |
| Poland | 600 | 5,600 | n/a |  | 0 | 19,800 | n/a |  | Poland | -46,800 | -7,600 | n/a |  | | -32,500 | | | -9,900 | n/a |
| Spain | 7,200 | 9,300 | -3,600 |  | 3,100 | 23,100 | 37,800 |  | Spain | -19,100 | -6,100 | -127,000 |  | | -22,700 | | | -1,300 | -40,000 |
| Sweden | 900 | 1,500 | 400 |  | 3,800 | 1,100 | 10,600 |  | Sweden | -3,900 | -3,400 | -20,800 |  | | -3,200 | | | -8,800 | -15,100 |
| UK | 41,200 | 11,900 | 46,200 |  | 45,000 | 21,400 | 122,400 |  | UK | 0 | -8000 | -112,500 |  | | 0 | | | -19,9100 | -58,500 |
| Total | 94,500 | 63,400 | 333,000 |  | 134,100 | 149,300 | 274,100 |  | Total | -157,100 | -76,00 | -609,400 |  | | -102,100 | | | -148,900 | -710,700 |

eTable 2: Country wise difference in cases of having any disease

|  | 1. Change in Abs Numbers with any disease (best practice) | | | | | | |  |  | 1. Change in Abs Numbers with any disease (worst practice) | | | | | | |
| --- | --- | --- | --- | --- | --- | --- | --- | --- | --- | --- | --- | --- | --- | --- | --- | --- |
|  |  | **Males** |  |  |  | **Females** |  |  |  |  | **Males** |  |  |  | **Females** |  |
|  | **Alcohol** | **BMI** | **Smoking** |  | **Alcohol** | **BMI** | **Smoking** |  |  | **Alcohol** | **BMI** | **Smoking** |  | **Alcohol** | **BMI** | **Smoking** |
| Denmark | -4350 | 250 | -11300 |  | 8250 | 150 | -17550 |  | Denmark | 6250 | 24650 | 450 |  | 8250 | 36200 | 300 |
| Finland | -2700 | -15300 | -5050 |  | 4750 | -19950 | -7400 |  | Finland | 7600 | 3100 | 9250 |  | 4350 | 5250 | 15100 |
| France | -57400 | -21200 | -7150 |  | 62100 | -84150 | 950 |  | France | 69300 | 218600 | 136250 |  | 80400 | 232900 | 245600 |
| Germany | -95350 | -236650 | -199750 |  | 85100 | -322650 | -24450 |  | Germany | 85150 | 64950 | 23050 |  | 79750 | 190050 | 376750 |
| Ireland | -2800 | -7700 | -200 |  | 1000 | -9850 | -7800 |  | Ireland | 3700 | 3350 | 10400 |  | 1950 | 4650 | 6950 |
| Italy | -75850 | -72400 | -38800 |  | -16250 | -234500 | -8200 |  | Italy | 31250 | 148800 | 110050 |  | 100 | 107050 | 280400 |
| Netherlands | -11200 | -7300 | -28750 |  | 10250 | -37250 | -41800 |  | Netherlands | 20400 | 63600 | 7350 |  | 11250 | 64350 | 19950 |
| Poland | 0 | -59200 | -35950 |  | 0 | -176250 | -168200 |  | Poland | 65800 | 66150 | 142500 |  | 2200 | 70100 | 116700 |
| Spain | -56250 | -87300 | -15200 |  | -12750 | -183550 | -57050 |  | Spain | 36250 | 93150 | 72150 |  | -10100 | 23800 | 108300 |
| Sweden | -1950 | -8150 | -600 |  | 13350 | -9100 | -17850 |  | Sweden | 14100 | 26300 | 26050 |  | 12400 | 47600 | 20300 |
| UK | -91950 | -140650 | -35950 |  | 1800 | -175100 | -168200 |  | UK | 0 | 35300 | 142500 |  | 0 | 58900 | 116700 |

eTable 3 Country wise overview of disease prevalence for included diseases.

| **Denmark** | **Breast Cancer** | | **Colorectal Cancer** | | **COPD** | | **Diabetes** | | **Eso Cancer** | | **IHD** | | **Lung Cancer** | | **Oral Cancer** | | **Stroke** | |
| --- | --- | --- | --- | --- | --- | --- | --- | --- | --- | --- | --- | --- | --- | --- | --- | --- | --- | --- |
|  | **M** | **F** | **M** | **F** | **M** | **F** | **M** | **F** | **M** | **F** | **M** | **F** | **M** | **F** | **M** | **F** | **M** | **F** |
| 0-15 | n/a | 0.00% | 0.00% | 0.00% | 0.00% | 0.00% | 0.00% | 0.00% | 0.00% | 0.00% | 0.00% | 0.00% | 0.00% | 0.00% | 0.00% | 0.00% | 0.00% | 0.00% |
| 16-30 | n/a | 0.00% | 0.00% | 0.00% | 0.00% | 0.00% | 0.00% | 0.10% | 0.00% | 0.00% | 0.00% | 0.00% | 0.00% | 0.00% | 0.00% | 0.00% | 0.00% | 0.00% |
| 31-45 | n/a | 0.30% | 0.00% | 0.00% | 0.10% | 0.10% | 1.70% | 2.30% | 0.00% | 0.00% | 0.20% | 0.10% | 0.00% | 0.00% | 0.00% | 0.00% | 0.20% | 0.10% |
| 46-60 | n/a | 2.10% | 0.30% | 0.30% | 1.30% | 1.40% | 6.70% | 5.00% | 0.00% | 0.00% | 2.90% | 1.40% | 0.10% | 0.10% | 0.20% | 0.10% | 1.60% | 0.80% |
| 61-75 | n/a | 5.00% | 1.30% | 1.10% | 4.80% | 4.70% | 13.40% | 10.00% | 0.00% | 0.00% | 10.10% | 6.00% | 0.30% | 0.30% | 0.60% | 0.20% | 5.60% | 3.30% |
| 76-95 | n/a | 7.60% | 3.30% | 2.50% | 6.10% | 4.50% | 15.30% | 13.70% | 0.10% | 0.00% | 18.40% | 13.40% | 0.60% | 0.40% | 0.90% | 0.40% | 13.20% | 9.80% |
|  |  |  |  |  |  |  |  |  |  |  |  |  |  |  |  |  |  |  |
| **Finland** | **Breast Cancer** | | **Colorectal Cancer** | | **COPD** | | **Diabetes** | | **Eso Cancer** | | **IHD** | | **Lung Cancer** | | **Oral Cancer** | | **Stroke** | |
|  | **M** | **F** | **M** | **F** | **M** | **F** | **M** | **F** | **M** | **F** | **M** | **F** | **M** | **F** | **M** | **F** | **M** | **F** |
| 0-15 | n/a | 0.00% | 0.00% | 0.00% | 0.00% | 0.00% | 0.00% | 0.00% | 0.00% | 0.00% | 0.00% | 0.00% | 0.00% | 0.00% | 0.00% | 0.00% | 0.00% | 0.00% |
| 16-30 | n/a | 0.00% | 0.00% | 0.00% | 0.00% | 0.00% | 0.00% | 0.00% | 0.00% | 0.00% | 0.00% | 0.00% | 0.00% | 0.00% | 0.00% | 0.00% | 0.00% | 0.00% |
| 31-45 | n/a | 0.30% | 0.00% | 0.10% | 0.10% | 0.10% | 0.60% | 0.40% | 0.00% | 0.00% | 0.40% | 0.20% | 0.00% | 0.00% | 0.00% | 0.00% | 0.20% | 0.10% |
| 46-60 | n/a | 2.10% | 0.20% | 0.30% | 1.10% | 1.00% | 4.10% | 2.20% | 0.00% | 0.00% | 5.20% | 2.60% | 0.10% | 0.00% | 0.10% | 0.10% | 1.50% | 0.70% |
| 61-75 | n/a | 5.40% | 0.90% | 0.90% | 4.00% | 3.20% | 10.20% | 7.30% | 0.00% | 0.00% | 19.50% | 12.00% | 0.40% | 0.10% | 0.40% | 0.20% | 6.20% | 3.80% |
| 76-95 | n/a | 8.00% | 2.50% | 2.10% | 5.70% | 3.60% | 12.30% | 11.90% | 0.10% | 0.00% | 36.90% | 27.00% | 0.90% | 0.30% | 0.80% | 0.40% | 15.00% | 11.70% |
|  |  |  |  |  |  |  |  |  |  |  |  |  |  |  |  |  |  |  |
| **France** | **Breast Cancer** | | **Colorectal Cancer** | | **COPD** | | **Diabetes** | | **Eso Cancer** | | **IHD** | | **Lung Cancer** | | **Oral Cancer** | | **Stroke** | |
|  | **M** | **F** | **M** | **F** | **M** | **F** | **M** | **F** | **M** | **F** | **M** | **F** | **M** | **F** | **M** | **F** | **M** | **F** |
| 0-15 | n/a | 0.00% | 0.00% | 0.00% | 0.00% | 0.00% | 0.00% | 0.00% | 0.00% | 0.00% | 0.00% | 0.00% | 0.00% | 0.00% | 0.00% | 0.00% | 0.00% | 0.00% |
| 16-30 | n/a | 0.00% | 0.00% | 0.00% | 0.00% | 0.00% | 0.00% | 0.00% | 0.00% | 0.00% | 0.00% | 0.00% | 0.00% | 0.00% | 0.00% | 0.00% | 0.00% | 0.00% |
| 31-45 | n/a | 0.40% | 0.00% | 0.00% | 0.00% | 0.10% | 1.20% | 1.00% | 0.00% | 0.00% | 0.10% | 0.00% | 0.00% | 0.00% | 0.10% | 0.00% | 0.10% | 0.10% |
| 46-60 | n/a | 2.60% | 0.40% | 0.30% | 1.00% | 0.90% | 6.40% | 4.10% | 0.00% | 0.00% | 1.50% | 0.60% | 0.10% | 0.00% | 0.60% | 0.10% | 1.10% | 0.40% |
| 61-75 | n/a | 6.30% | 1.80% | 1.10% | 4.00% | 2.60% | 15.20% | 9.50% | 0.20% | 0.00% | 5.90% | 3.10% | 0.40% | 0.10% | 1.60% | 0.30% | 4.30% | 2.40% |
| 76-95 | n/a | 8.80% | 4.30% | 2.60% | 5.20% | 2.40% | 16.20% | 11.00% | 0.40% | 0.00% | 10.50% | 6.70% | 0.60% | 0.10% | 2.40% | 0.40% | 10.10% | 7.20% |

|  |  |  |  |  |  |  |  |  |  |  |  |  |  |  |  |  |  |  |
| --- | --- | --- | --- | --- | --- | --- | --- | --- | --- | --- | --- | --- | --- | --- | --- | --- | --- | --- |
| **Germany** | **Breast Cancer** | | **Colorectal Cancer** | | **COPD** | | **Diabetes** | | **Eso Cancer** | | **IHD** | | **Lung Cancer** | | **Oral Cancer** | | **Stroke** | |
|  | **M** | **F** | **M** | **F** | **M** | **F** | **M** | **F** | **M** | **F** | **M** | **F** | **M** | **F** | **M** | **F** | **M** | **F** |
| 0-15 | n/a | 0.00% | 0.00% | 0.00% | 0.00% | 0.00% | 0.00% | 0.00% | 0.00% | 0.00% | 0.00% | 0.00% | 0.00% | 0.00% | 0.00% | 0.00% | 0.00% | 0.00% |
| 16-30 | n/a | 0.00% | 0.00% | 0.00% | 0.00% | 0.00% | 0.00% | 0.00% | 0.00% | 0.00% | 0.00% | 0.00% | 0.00% | 0.00% | 0.00% | 0.00% | 0.00% | 0.00% |
| 31-45 | n/a | 0.30% | 0.00% | 0.00% | 0.10% | 0.10% | 1.30% | 1.30% | 0.00% | 0.00% | 0.30% | 0.10% | 0.00% | 0.00% | 0.00% | 0.00% | 0.10% | 0.10% |
| 46-60 | n/a | 1.80% | 0.40% | 0.30% | 1.00% | 0.90% | 5.60% | 5.70% | 0.00% | 0.00% | 3.60% | 1.70% | 0.10% | 0.00% | 0.30% | 0.10% | 1.00% | 0.50% |
| 61-75 | n/a | 4.40% | 1.80% | 1.10% | 3.80% | 2.50% | 12.60% | 12.70% | 0.10% | 0.00% | 14.50% | 8.40% | 0.70% | 0.10% | 0.70% | 0.20% | 5.00% | 3.00% |
| 76-95 | n/a | 6.50% | 4.20% | 2.80% | 5.50% | 2.40% | 13.60% | 13.20% | 0.20% | 0.00% | 27.10% | 19.10% | 1.40% | 0.20% | 1.00% | 0.30% | 13.80% | 10.70% |
|  |  |  |  |  |  |  |  |  |  |  |  |  |  |  |  |  |  |  |
| **Ireland** | **Breast Cancer** | | **Colorectal Cancer** | | **COPD** | | **Diabetes** | | **Eso Cancer** | | **IHD** | | **Lung Cancer** | | **Oral Cancer** | | **Stroke** | |
|  | **M** | **F** | **M** | **F** | **M** | **F** | **M** | **F** | **M** | **F** | **M** | **F** | **M** | **F** | **M** | **F** | **M** | **F** |
| 0-15 | n/a | 0.00% | 0.00% | 0.00% | 0.00% | 0.00% | 0.00% | 0.00% | 0.00% | 0.00% | 0.00% | 0.00% | 0.00% | 0.00% | 0.00% | 0.00% | 0.00% | 0.00% |
| 16-30 | n/a | 0.00% | 0.00% | 0.00% | 0.00% | 0.00% | 0.00% | 0.00% | 0.00% | 0.00% | 0.00% | 0.00% | 0.00% | 0.00% | 0.00% | 0.00% | 0.00% | 0.00% |
| 31-45 | n/a | 0.30% | 0.00% | 0.00% | 0.10% | 0.10% | 0.90% | 1.00% | 0.00% | 0.00% | 0.30% | 0.10% | 0.00% | 0.00% | 0.00% | 0.00% | 0.10% | 0.10% |
| 46-60 | n/a | 2.00% | 0.30% | 0.30% | 1.10% | 1.10% | 4.10% | 3.20% | 0.00% | 0.00% | 4.20% | 2.00% | 0.10% | 0.10% | 0.10% | 0.00% | 1.10% | 0.90% |
| 61-75 | n/a | 4.80% | 1.70% | 1.20% | 3.70% | 3.90% | 8.40% | 6.40% | 0.10% | 0.00% | 16.30% | 9.50% | 0.40% | 0.20% | 0.40% | 0.10% | 5.00% | 4.00% |
| 76-95 | n/a | 6.90% | 4.10% | 2.80% | 5.10% | 3.60% | 9.20% | 8.10% | 0.10% | 0.10% | 30.00% | 21.20% | 0.80% | 0.40% | 0.60% | 0.20% | 13.40% | 11.30% |
|  |  |  |  |  |  |  |  |  |  |  |  |  |  |  |  |  |  |  |
| **Italy** | **Breast Cancer** | | **Colorectal Cancer** | | **COPD** | | **Diabetes** | | **Eso Cancer** | | **IHD** | | **Lung Cancer** | | **Oral Cancer** | | **Stroke** | |
|  | **M** | **F** | **M** | **F** | **M** | **F** | **M** | **F** | **M** | **F** | **M** | **F** | **M** | **F** | **M** | **F** | **M** | **F** |
| 0-15 | n/a | 0.00% | 0.00% | 0.00% | 0.00% | 0.00% | 0.00% | 0.00% | 0.00% | 0.00% | 0.00% | 0.00% | 0.00% | 0.00% | 0.00% | 0.00% | 0.00% | 0.00% |
| 16-30 | n/a | 0.00% | 0.00% | 0.00% | 0.00% | 0.00% | 0.00% | 0.10% | 0.00% | 0.00% | 0.00% | 0.00% | 0.00% | 0.00% | 0.00% | 0.00% | 0.00% | 0.00% |
| 31-45 | n/a | 0.40% | 0.00% | 0.10% | 0.00% | 0.10% | 1.00% | 0.90% | 0.00% | 0.00% | 0.20% | 0.10% | 0.00% | 0.00% | 0.00% | 0.00% | 0.10% | 0.00% |
| 46-60 | n/a | 2.60% | 0.40% | 0.40% | 1.20% | 0.90% | 4.20% | 3.60% | 0.00% | 0.00% | 2.50% | 1.20% | 0.10% | 0.10% | 0.20% | 0.10% | 1.00% | 0.50% |
| 61-75 | n/a | 5.90% | 2.00% | 1.40% | 4.60% | 2.60% | 12.20% | 12.50% | 0.10% | 0.00% | 9.80% | 5.90% | 0.70% | 0.30% | 0.60% | 0.20% | 5.20% | 3.30% |
| 76-95 | n/a | 8.60% | 4.90% | 3.20% | 5.50% | 2.50% | 17.50% | 18.10% | 0.10% | 0.00% | 17.50% | 12.80% | 1.30% | 0.50% | 1.00% | 0.30% | 15.80% | 12.30% |

|  |  |  |  |  |  |  |  |  |  |  |  |  |  |  |  |  |  |  |
| --- | --- | --- | --- | --- | --- | --- | --- | --- | --- | --- | --- | --- | --- | --- | --- | --- | --- | --- |
| **Netherlands** | **Breast Cancer** | | **Colorectal Cancer** | | **COPD** | | **Diabetes** | | **Eso Cancer** | | **IHD** | | **Lung Cancer** | | **Oral Cancer** | | **Stroke** | |
|  | **M** | **F** | **M** | **F** | **M** | **F** | **M** | **F** | **M** | **F** | **M** | **F** | **M** | **F** | **M** | **F** | **M** | **F** |
| 0-15 | n/a | 0.00% | 0.00% | 0.00% | 0.00% | 0.00% | 0.00% | 0.00% | 0.00% | 0.00% | 0.00% | 0.00% | 0.00% | 0.00% | 0.00% | 0.00% | 0.00% | 0.00% |
| 16-30 | n/a | 0.00% | 0.00% | 0.00% | 0.00% | 0.00% | 0.00% | 0.00% | 0.00% | 0.00% | 0.00% | 0.00% | 0.00% | 0.00% | 0.00% | 0.00% | 0.00% | 0.00% |
| 31-45 | n/a | 0.50% | 0.00% | 0.00% | 0.10% | 0.10% | 1.30% | 1.00% | 0.00% | 0.00% | 0.30% | 0.10% | 0.00% | 0.00% | 0.00% | 0.00% | 0.10% | 0.10% |
| 46-60 | n/a | 2.60% | 0.30% | 0.30% | 1.50% | 1.90% | 5.70% | 4.20% | 0.00% | 0.00% | 3.50% | 1.60% | 0.10% | 0.10% | 0.20% | 0.10% | 0.90% | 0.70% |
| 61-75 | n/a | 5.70% | 1.70% | 1.30% | 5.00% | 4.80% | 12.70% | 11.70% | 0.10% | 0.00% | 13.60% | 7.80% | 0.40% | 0.20% | 0.50% | 0.30% | 4.50% | 3.50% |
| 76-95 | n/a | 8.60% | 4.10% | 3.00% | 6.60% | 5.30% | 15.90% | 17.40% | 0.10% | 0.00% | 28.20% | 18.90% | 0.80% | 0.20% | 0.90% | 0.40% | 12.60% | 10.10% |
|  |  |  |  |  |  |  |  |  |  |  |  |  |  |  |  |  |  |  |
| **Poland** | **Breast Cancer** | | **Colorectal Cancer** | | **COPD** | | **Diabetes** | | **Eso Cancer** | | **IHD** | | **Lung Cancer** | | **Oral Cancer** | | **Stroke** | |
|  | **M** | **F** | **M** | **F** | **M** | **F** | **M** | **F** | **M** | **F** | **M** | **F** | **M** | **F** | **M** | **F** | **M** | **F** |
| 0-15 | n/a | 0.00% | 0.00% | 0.00% | 0.00% | 0.00% | 0.00% | 0.00% | 0.00% | 0.00% | 0.00% | 0.00% | 0.00% | 0.00% | 0.00% | 0.00% | 0.00% | 0.00% |
| 16-30 | n/a | 0.00% | 0.00% | 0.00% | 0.00% | 0.00% | 0.10% | 0.10% | 0.00% | 0.00% | 0.00% | 0.00% | 0.00% | 0.00% | 0.00% | 0.00% | 0.00% | 0.00% |
| 31-45 | n/a | 0.20% | 0.00% | 0.00% | 0.50% | 0.70% | 3.20% | 3.20% | 0.00% | 0.00% | 0.30% | 0.10% | 0.00% | 0.00% | 0.00% | 0.00% | 0.10% | 0.10% |
| 46-60 | n/a | 1.20% | 0.20% | 0.20% | 2.30% | 2.20% | 8.20% | 9.50% | 0.00% | 0.00% | 3.50% | 1.80% | 0.10% | 0.10% | 0.10% | 0.10% | 1.60% | 0.90% |
| 61-75 | n/a | 3.10% | 0.80% | 0.70% | 8.80% | 4.90% | 12.50% | 16.10% | 0.00% | 0.00% | 14.30% | 9.40% | 0.40% | 0.20% | 0.40% | 0.10% | 7.60% | 4.90% |
| 76-95 | n/a | 4.40% | 1.70% | 1.10% | 8.80% | 4.90% | 12.60% | 16.70% | 0.00% | 0.00% | 25.20% | 19.20% | 0.50% | 0.40% | 0.70% | 0.20% | 15.00% | 11.70% |
|  |  |  |  |  |  |  |  |  |  |  |  |  |  |  |  |  |  |  |
| **Spain** | **Breast Cancer** | | **Colorectal Cancer** | | **COPD** | | **Diabetes** | | **Eso Cancer** | | **IHD** | | **Lung Cancer** | | **Oral Cancer** | | **Stroke** | |
|  | **M** | **F** | **M** | **F** | **M** | **F** | **M** | **F** | **M** | **F** | **M** | **F** | **M** | **F** | **M** | **F** | **M** | **F** |
| 0-15 | n/a | 0.00% | 0.00% | 0.00% | 0.00% | 0.00% | 0.00% | 0.00% | 0.00% | 0.00% | 0.00% | 0.00% | 0.00% | 0.00% | 0.00% | 0.00% | 0.00% | 0.00% |
| 16-30 | n/a | 0.00% | 0.00% | 0.00% | 0.00% | 0.00% | 0.10% | 0.10% | 0.00% | 0.00% | 0.00% | 0.00% | 0.00% | 0.00% | 0.00% | 0.00% | 0.00% | 0.00% |
| 31-45 | n/a | 0.30% | 0.00% | 0.00% | 0.00% | 0.10% | 1.70% | 1.60% | 0.00% | 0.00% | 0.10% | 0.10% | 0.00% | 0.00% | 0.10% | 0.00% | 0.10% | 0.00% |
| 46-60 | n/a | 1.70% | 0.30% | 0.20% | 1.20% | 1.10% | 7.50% | 4.90% | 0.00% | 0.00% | 1.90% | 0.90% | 0.20% | 0.00% | 0.40% | 0.10% | 1.00% | 0.50% |
| 61-75 | n/a | 3.80% | 1.40% | 0.90% | 4.00% | 3.60% | 18.60% | 14.10% | 0.10% | 0.00% | 7.60% | 4.70% | 0.60% | 0.10% | 1.20% | 0.20% | 5.00% | 3.00% |
| 76-95 | n/a | 5.40% | 3.30% | 1.90% | 4.40% | 3.90% | 16.00% | 17.50% | 0.10% | 0.00% | 13.30% | 10.00% | 0.90% | 0.10% | 2.00% | 0.30% | 12.60% | 9.80% |
|  |  |  |  |  |  |  |  |  |  |  |  |  |  |  |  |  |  |  |

| **Sweden** | **Breast Cancer** | | **Colorectal Cancer** | | **COPD** | | **Diabetes** | | **Eso Cancer** | | **IHD** | | **Lung Cancer** | | **Oral Cancer** | | **Stroke** | |
| --- | --- | --- | --- | --- | --- | --- | --- | --- | --- | --- | --- | --- | --- | --- | --- | --- | --- | --- |
|  | **M** | **F** | **M** | **F** | **M** | **F** | **M** | **F** | **M** | **F** | **M** | **F** | **M** | **F** | **M** | **F** | **M** | **F** |
| 0-15 | n/a | 0.00% | 0.00% | 0.00% | 0.00% | 0.00% | 0.00% | 0.00% | 0.00% | 0.00% | 0.00% | 0.00% | 0.00% | 0.00% | 0.00% | 0.00% | 0.00% | 0.00% |
| 16-30 | n/a | 0.00% | 0.00% | 0.00% | 0.00% | 0.00% | 0.10% | 0.10% | 0.00% | 0.00% | 0.00% | 0.00% | 0.00% | 0.00% | 0.00% | 0.00% | 0.00% | 0.00% |
| 31-45 | n/a | 0.30% | 0.00% | 0.00% | 0.00% | 0.10% | 1.90% | 1.40% | 0.00% | 0.00% | 0.30% | 0.10% | 0.00% | 0.00% | 0.00% | 0.00% | 0.10% | 0.00% |
| 46-60 | n/a | 2.10% | 0.20% | 0.20% | 1.20% | 1.30% | 6.00% | 3.80% | 0.00% | 0.00% | 3.90% | 1.90% | 0.10% | 0.10% | 0.10% | 0.10% | 1.00% | 0.60% |
| 61-75 | n/a | 5.30% | 1.10% | 1.00% | 4.30% | 4.00% | 12.70% | 9.60% | 0.00% | 0.00% | 14.20% | 8.30% | 0.20% | 0.20% | 0.30% | 0.20% | 4.80% | 3.00% |
| 76-95 | n/a | 8.00% | 3.00% | 2.40% | 5.00% | 3.70% | 16.40% | 14.50% | 0.10% | 0.00% | 27.00% | 19.20% | 0.30% | 0.10% | 0.70% | 0.40% | 14.10% | 10.70% |
|  |  |  |  |  |  |  |  |  |  |  |  |  |  |  |  |  |  |  |
| **UK** | **Breast Cancer** | | **Colorectal Cancer** | | **COPD** | | **Diabetes** | | **Eso Cancer** | | **IHD** | | **Lung Cancer** | | **Oral Cancer** | | **Stroke** | |
|  | **M** | **F** | **M** | **F** | **M** | **F** | **M** | **F** | **M** | **F** | **M** | **F** | **M** | **F** | **M** | **F** | **M** | **F** |
| 0-15 | n/a | 0.00% | 0.00% | 0.00% | 0.00% | 0.00% | 0.00% | 0.00% | 0.00% | 0.00% | 0.00% | 0.00% | 0.00% | 0.00% | 0.00% | 0.00% | 0.00% | 0.00% |
| 16-30 | n/a | 0.00% | 0.00% | 0.00% | 0.00% | 0.00% | 0.00% | 0.00% | 0.00% | 0.00% | 0.00% | 0.00% | 0.00% | 0.00% | 0.00% | 0.00% | 0.00% | 0.00% |
| 31-45 | n/a | 0.30% | 0.00% | 0.00% | 0.00% | 0.10% | 0.70% | 0.80% | 0.00% | 0.00% | 0.30% | 0.10% | 0.00% | 0.00% | 0.00% | 0.00% | 0.10% | 0.10% |
| 46-60 | n/a | 2.10% | 0.20% | 0.20% | 0.80% | 0.80% | 4.00% | 2.80% | 0.00% | 0.00% | 3.90% | 1.90% | 0.10% | 0.10% | 0.10% | 0.10% | 1.10% | 0.70% |
| 61-75 | n/a | 5.00% | 1.40% | 1.00% | 3.40% | 2.80% | 9.90% | 6.90% | 0.10% | 0.00% | 14.90% | 8.60% | 0.50% | 0.30% | 0.40% | 0.20% | 5.20% | 3.90% |
| 76-95 | n/a | 7.60% | 3.70% | 2.60% | 5.90% | 3.70% | 11.90% | 9.10% | 0.10% | 0.10% | 27.70% | 19.50% | 1.30% | 0.70% | 0.70% | 0.30% | 13.80% | 12.40% |

eTable 4: Overview of relative risks from alcohol to diseases and total mortality (below the age of 15 all relative risks are set 1)

| Outcome | Males aged 15 years and over | | | | |  | Females aged 15 years and over | | | | |
| --- | --- | --- | --- | --- | --- | --- | --- | --- | --- | --- | --- |
|  | Drinking categories (grams per day) | | | | |  | Drinking categories (grams per day) | | | | |
|  | 0 - <0.25 | 0.25 - <20 | 20 - <40 | 40 - <60 | ≥60 |  | 0 - <0.25 | 0.25 - <20 | 20 - <40 | 40 - <60 | ≥60 |
| All-cause mortality |  |  |  |  |  |  |  |  |  |  |  |
| Persons Aged 16-24  Persons Aged 25-34  Persons Aged 35-44  Persons Aged 45-54  Persons Aged 55-64  Persons Aged 65-74  Persons Aged 75-84  Persons Aged 85-95 | 1.00  1.00  1.00  1.00  1.00  1.00  1.00  1.00 | 1.07  1.05  1.00  0.96  0.94  0.94  0.95  0.96 | 1.25  1.21  1.10  1.01  0.98  0.97  0.97  0.98 | 1.48  1.40  1.23  1.10  1.04  1.02  1.02  1.02 | 1.88  1.75  1.47  1.26  1.16  1.11  1.11  1.09 |  | 1.00  1.00  1.00  1.00  1.00  1.00  1.00  1.00 | 1.04  1.04  1.03  1.02  1.00  0.99  0.98  0.98 | 1.17  1.15  1.15  1.13  1.09  1.06  1.05  1.03 | 1.31  1.29  1.30  1.26  1.22  1.17  1.15  1.12 | 1.58  1.54  1.56  1.51  1.46  1.38  1.35  1.27 |
| IHD | 1.00 | 0.82 | 0.82 | 0.87 | 1.13 |  | 1.00 | 0.82 | 0.82 | 0.87 | 1.13 |
| Stroke | 1.00 | 0.91 | 1.01 | 1.18 | 1.55 |  | 1.00 | 0.7 | 0.79 | 1.08 | 2.74 |
| Diabetes mellitus | 1.00 | 0.72 | 0.86 | 1.00 | 1.00 |  | 1.00 | 0.72 | 0.86 | 1.00 | 1.00 |
| COPD | 1.00 | 1.00 | 1.00 | 1.00 | 1.00 |  | 1.00 | 1.00 | 1.00 | 1.00 | 1.00 |
| Lung cancer | 1.00 | 1.00 | 1.00 | 1.00 | 1.00 |  | 1.00 | 1.00 | 1.00 | 1.00 | 1.00 |
| Colorectal cancer | 1.00 | 1.00 | 1.08 | 1.30 | 1.72 |  | 1.00 | 1.00 | 1.11 | 1.33 | 1.62 |
| Oral cancer | 1.00 | 1.31 | 2.08 | 3.02 | 4.32 |  | 1.00 | 1.33 | 2.18 | 3.26 | 4.85 |
| Breast cancer | 1.00 | 1.00 | 1.00 | 1.00 | 1.00 |  | 1.00 | 1.00 | 1.23 | 1.42 | 1.68 |
| Esophageal cancer | 1.00 | 1.17 | 1.61 | 2.19 | 3.18 |  | 1.00 | 1.17 | 1.61 | 2.19 | 3.18 |
| Rehm J, Sulkowska U, Mańczuk M, Boffetta P, Powles J, Popova S, Zatoński W. Alcohol accounts for a high proportion of premature mortality in central and eastern Europe. Int J Epidemiol. 2007 Apr;36(2):458-67. Epub 2007 Jan 24.  White IR, Altmann DR, Nanchahal K. ‘Optimal’ levels of alcohol consumption for men and women at different ages, and the all-cause mortality attributable to drinking. London: London School of Hygiene and Tropical Medicine, 2000.[Technical Report]  White IR, Altmann DR, Nanchahal K. Alcohol consumption and mortality: modelling risks for men and women at different ages. British Medical Journal 2002; 325:191-194.  Tabak C, Smit HA, Räsänen L, Fidanza F, Menotti A, Nissinen A, Feskens EJ, Heederik D, Kromhout D. : Alcohol consumption in relation to 20-year COPD mortality and pulmonary function in middle-aged men from three European countries. Epidemiology. 2001; 12:239-245. | | | | | | | | | | | |
| Further details available on the data reports on [www.dynamo-hia.eu](http://www.dynamo-hia.eu) | | | | | | | | | | | |

eTable 5: Overview of relative risks from smoking to diseases and total mortality used in the example applications (below the age of 35 all relative risks are set to 1)

| Outcome | Male aged 35 and above | | |  | Female aged 35 and above | | |
| --- | --- | --- | --- | --- | --- | --- | --- |
|  | Never Smoker | Current Smoker | Former Smoker |  | Never Smoker | Current Smoker | Former Smoker |
| All-cause mortality |  |  |  |  |  |  |  |
| Persons Aged 35–39    Persons Aged 40-44    Persons Aged 45–49    Persons Aged 50–54    Persons Aged 55–59    Persons Aged 60-64    Persons Aged 65+ | 1.00  1.00  1.00  1.00  1.00  1.00  1.00 | 2.07  2.07  2.07  2.07  2.07  2.07  2.07 | 1.35  1.35  1.35  1.35  1.35  1.35  1.35 |  | 1.00  1.00  1.00  1.00  1.00  1.00  1.00 | 1.74  1.74  1.74  1.74  1.74  1.74  1.74 | 1.23  1.23  1.23  1.23  1.23  1.23  1.23 |
| Lip, Oral Cavity, Pharynx Cancers |  |  |  |  |  |  |  |
| Persons Aged 35–39    Persons Aged 40-44    Persons Aged 45–49    Persons Aged 50–54    Persons Aged 55–59    Persons Aged 60-64    Persons Aged 65+ | 1.00  1.00  1.00  1.00  1.00  1.00  1.00 | 10.89  10.89  10.89  10.89  10.89  10.89  10.89 | 3.40  3.40  3.40  3.40  3.40  3.40  3.40 |  | 1.00  1.00  1.00  1.00  1.00  1.00  1.00 | 5.08  5.08  5.08  5.08  5.08  5.08  5.08 | 2.29  2.29  2.29  2.29  2.29  2.29  2.29 |
| Esophagus Cancer |  |  |  |  |  |  |  |
| Persons Aged 35–39    Persons Aged 40-44    Persons Aged 45–49    Persons Aged 50–54    Persons Aged 55–59    Persons Aged 60-64    Persons Aged 65+ | 1.00  1.00  1.00  1.00  1.00  1.00  1.00 | 6.76  6.76  6.76  6.76  6.76  6.76  6.76 | 4.46  4.46  4.46  4.46  4.46  4.46  4.46 |  | 1.00  1.00  1.00  1.00  1.00  1.00  1.00 | 7.75  7.75  7.75  7.75  7.75  7.75  7.75 | 2.79  2.79  2.79  2.79  2.79  2.79  2.79 |
| Lung cancer |  |  |  |  |  |  |  |
| Persons Aged 35–39    Persons Aged 40-44    Persons Aged 45–49    Persons Aged 50–54    Persons Aged 55–59    Persons Aged 60-64    Persons Aged 65+ | 1.00  1.00  1.00  1.00  1.00  1.00  1.00 | 1.30  1.00  5.78  24.97  34.02  31.47  28.40 | 1.00  1.00  2.37  10.70  11.66  11.71  9.70 |  | 1.00  1.00  1.00  1.00  1.00  1.00  1.00 | 2.00  1.00  18.08  11.14  17.87  13.32  17.49 | 1.00  1.00  8.07  3.28  5.33  4.91  5.54 |
| IHD |  |  |  |  |  |  |  |
| Persons Aged 35–39    Persons Aged 40-44    Persons Aged 45–49    Persons Aged 50–54    Persons Aged 55–59    Persons Aged 60-64    Persons Aged 65+ | 1.00  1.00  1.00  1.00  1.00  1.00  1.00 | 3.25  4.71  5.85  3.69  2.71  2.39  1.91 | 1.21  1.15  2.03  1.93  1.64  1.58  1.40 |  | 1.00  1.00  1.00  1.00  1.00  1.00  1.00 | 1.00  1.89  7.71  5.69  3.06  2.56  2.48 | 1.44  2.25  2.08  2.95  1.19  1.08  1.22 |
| Stroke |  |  |  |  |  |  |  |
| Persons Aged 35–39    Persons Aged 40-44    Persons Aged 45–49    Persons Aged 50–54    Persons Aged 55–59    Persons Aged 60-64    Persons Aged 65+ | 1.00  1.00  1.00  1.00  1.00  1.00  1.00 | 1.00  1.05  3.75  6.08  3.96  2.55  2.69 | 1.00  1.00  1.00  2.24  1.14  1.01  1.29 |  | 1.00  1.00  1.00  1.00  1.00  1.00  1.00 | 2.00  5.67  8.22  4.58  5.77  2.76  2.58 | 1.00  2.25  1.19  1.38  1.22  1.28  1.14 |
| COPD |  |  |  |  |  |  |  |
| Persons Aged 35–39    Persons Aged 40-44    Persons Aged 45–49    Persons Aged 50–54    Persons Aged 55–59    Persons Aged 60-64    Persons Aged 65+ | 1.00  1.00  1.00  1.00  1.00  1.00  1.00 | 1.00  1.00  1.00  8.13  9.80  13.21  18.93 | 1.00  1.00  1.00  3.06  8.25  12.65  11.92 |  | 1.00  1.00  1.00  1.00  1.00  1.00  1.00 | 1.00  1.00  1.00  12.92  9.47  11.19  14.72 | 1.00  1.00  1.00  7.39  5.55  6.63  9.73 |
| References:  Ellison LF et al. Health consequences of smoking among Canadian smokers: An update. Chronic Dis Can 1999; 20:36-9.  American Cancer Society´s Cancer Prevention Study II age-specific relative risks (1982-1988).  American Cancer Society´s Cancer Prevention Study II age-specific relative risks (1982-1988).  Tanuseputro P, Manuel DG, Schultz SE, Johansen H, Mustard CA. Improving population attributable fraction methods: examining smoking-attributable mortality for 87 geographic regions in Canada. Am J Epidemiol. 2005 Apr 15;161(8):787-98 | | | | | | | |
| Further details available on the data reports on [www.dynamo-hia.eu](http://www.dynamo-hia.eu) | | | | | | | |

eTable 6: Table of estimated relative risks of disease linked to overweight and obesity

|  | males | |  | females | |  | Age adjustments**  (multiplier of differential risk) |
| --- | --- | --- | --- | --- | --- | --- | --- |
|  | overweight  BMI 25-29.9 | obesity  BMI 30 or more |  | overweight  BMI 25-29.9 | besity  BMI 30 or more |  |  |
| All cause mortality | 1.20 | 1.55 |  | 1.15 | 1.50 |  | x 0.98 from age 50  x 0.95 from age 60  x 0.90 from age 70 |
| IHD | 1.35 | 2.00 |  | 1.35 | 2.00 |  | x 0.70 age over 65 |
| Stroke | 1.20 | 1.50 |  | 1.20 | 1.55 |  | x 0.75 from age 65 |
| Diabetes | 2.25 | 5.50 |  | 2.30 | 7.00 |  | x 0.92 from age 60  x 0.90 from age 75 |
| COPD | 1.00 | 1.00 |  | 1.00 | 1.00 |  |  |
| Lung cancer | 0.80 | 0.65 |  | 0.88 | 0.70 |  |  |
| Breast cancer | 1.00 | 1.00 |  | 1.00  1.12 over age 50 | 1.00  1.25 over age 50 |  |  |
| Oral cancer | 0.80 | 0.65 |  | 0.88 | 0.70 |  |  |
| Colorectal cancer | 1.20 | 1.40 |  | 1.08 | 1.10 |  | x 0.90 from age 45 |
| Eso cancer | 1.00 | 1.00 |  | 1.00 | 1.00 |  |  |

* Adjustments for age are given as multipliers of the differential risk from the base (1.0).

Thus an adjustment multiplier of x0.95 applied to an RR of 1.20 would lead to an RR of 1.19

The relative risk estimates for obesity are obtained from a review conducted by the International Association for the Study of Obesity, and are based on a literature search of systematic reviews and meta-analyses published up to the end of 2009. Full details are given at <http://www.iaso.org/policy/healthimpactobesity/>.

eTable 7: Overview of relative risks from diabetes to IHD and stroke used in the example applications

|  | Males | Females |
| --- | --- | --- |
| Diabetes to IHD |  |  |
| Persons Aged up to 55    Persons Aged 56+ | 2.66  1.93 | 3.53  2.59 |
|  |  |  |
| Diabetes to stroke |  |  |
| Persons Aged up to 49    Persons Aged 50+ | 2.00  1.80 | 2.90  2.20 |
| Yusuf S, Hawken S et al. Effect of potentially modifiable risk factors associated with myocardial infarction in 52 countries (the INTERHEART study): case-control study. 2004; 364: 937- 52.  Barrett-Connor E, Khaw KT. Diabetes mellitus: an independent risk factor for stroke? Am J Epidemiol. 1988 Jul;128(1):116-23. Gu K, Cowie CC, Harris MI. Mortality in adults with and without diabetes in a national cohort of the U.S. population, 1971-1993. Diabetes Care. 1998Jul;21(7):1138-45. | | |
| Further details available on the data reports on [www.dynamo-hia.eu](http://www.dynamo-hia.eu) | | |

## eAppendix: Model Core of DYNAMO-HIA

In the following we outline in detail the model core of DYNAMO-HIA^[[1]](#footnote-1)^. DYNAMO-HIA is Markov-type model based on a multi-state model (MSM). The change of the state depends only on current characteristics (i.e. age, sex, risk-factor status, and health status). The MSM is implemented as a *partial micro-simulation* combining a stochastic micro-simulation to project risk-factor behavior with a deterministic macro approach for the disease life table ^1^. In the micro-simulation module large numbers of distinctive risk-factor biographies are simulated: Given the age and sex-specific transition probabilities between risk-factor states, the risk-factor status of each simulated individual is updated in annual increments (see Fig. 1 for details). In the macro module, as many disease life tables are constructed as there are risk-factor biographies. These disease life tables account for competing risks and multiple morbidity ^2^. The exact configuration of the disease life tables, i.e. the number and kind of diseases, can be specified by the user (see Fig. 2 for details). For every risk-factor biography, the probability of disease incidence and mortality over time is calculated, accounting for the current age, risk-factor, and disease status (see Fig. 3 for details). These biography-specific life tables are calculated for each birth-cohort, i.e. all individuals that are born in the same calendar year. For example, for a cohort of newborns first the risk-factor biographies are projected and then disease life tables are calculated. Older cohorts, i.e. born before the first simulation year, already start out having the disease prevalence as specified by the input data, which is then similarly updated. Population values are obtained by aggregating the individual biography/diseases life tables: either across cohorts at a given simulation time point to obtain period measures or along cohorts to obtain cohort specific measures (see Fig. 4 for details). The split into a micro and a macro module is done purely for computational convenience and micro- and macro-simulations yield the same result when used with the same data ^3, 4^. However, time and memory requirements in macro-simulations rise exponentially when the number of simulated states increases and micro-simulations – unlike customary multi-state life tables – do not require the a priori specification of all theoretically possible combinations of diseases/risk-factor states, but only those states that are actually occupied. But for simulating rare events – e.g. lung cancer at young ages – micro-simulations require the simulation of large numbers of individuals, offsetting the savings in time and memory requirements.

The epidemiological model uses relative risks by risk-factor class, i.e. incidences in exposed risk-factor classes are a multiple of the incidence in non-exposed. The total mortality, i.e. population level mortality by age and sex, is being decomposed in the mortality due to the diseases included in the model and other cause mortality. This decomposition assumes additive mortality: the total mortality rate in the population is explained as the sum of the mortality rate of the included diseases and other-cause mortality, i.e. mortality from all causes/diseases that are not explicitly included in the model.


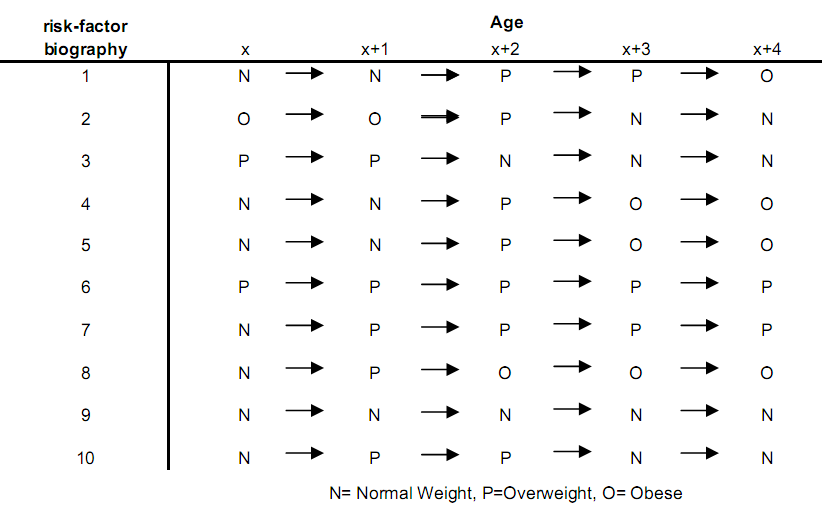


Fig. 1: Example of risk-factor biographies for a risk-factor with three categories. DYNAMO-HIA simulates individuals and projects their risk-factor biographies. The risk-factor status is being updated in one-year increments, given age- and sex-specific transition probabilities. The age- and sex-specific risk-factor status determines the relative risk of a person to contract a disease or to die. DYNAMO-HIA allows one risk-factor per scenario. This risk-factor can be either categorical (up to ten categories), duration dependent (up to ten categories of which one is duration dependent, i.e. the risk on disease in this category depends on how long a person is in the category), or a continuous distribution (normal or log-normal, specified by entering mean, standard deviation, and, in the case of the log-normal, skewness).

Fig. 2: Stylized structure of disease life table. The disease life tables contain diseases clusters. Each disease cluster consists of one or more diseases. Within disease clusters, intermediate diseases - that increase the risk of getting another disease - can be specified (e.g. having diabetes increases the risk of getting IHD). All diseases are chronic diseases, i.e. excess mortality depends on age and sex and not on time since onset of disease. However, acutely fatal and/or cured fraction can be specified for diseases. The disease life table assumes independence between disease clusters. The user can freely specify the relative risks from risk-factor to disease, from risk-factor to death, and from intermediate disease to other diseases.

Fig. 3: Stylized cohort life tables (with only one disease, three different biographies, and five time steps). For every risk-factor biography, a disease life table is constructed. Diseases incidence, i.e. transition from healthy to a disease, equals the baseline incidence, i.e. incidence when in a risk-factor class with a relative risk of one for the specific age- and sex-category, times the relative risk due to the given risk-factor and diseases status (in the case of an intermediate disease). The transition from healthy to dead equals the baseline other-cause mortality of the healthy, i.e. age- and sex-specific total mortality rate minus the excess mortality rate of the diseases included in the disease life table, multiplied by the relative risk due to the given risk-factor status on other cause mortality. The transition from diseased to dead equals the sum of the excess mortality of the disease (given each and sex) and the baseline other cause mortality of the healthy, multiplied by the relative risk in the given risk-factor status. Remission is not explicitly modeled, but for *diseases with cured fraction* the excess mortality is zero in a “cured”, i.e. user-specified, fraction. *Partly acutely fatal diseases*, i.e. diseases with very high mortality immediately after contracting the disease while for those who survive this critical period the excess mortality only depends on age and sex, are modeled by specifying the fraction of the incidence cases that die immediately.

Fig. 4: Schematic overview of the dimension of a multi-cohort, multistate-life table. Each plane is a distinct cohort with varying starting ages for cohorts already existing at the starting year of the simulation and starting age zero for cohorts born during the simulation run. The cohort life tables, consisting of the set of individual risk-factor biographies, follow every already existing birth cohort until the cohort reaches 105 years of age. In addition, every year of the simulation a cohort of newborns is created and – after simulating individual risk-factor biographies for them – is followed through the appropriate disease life tables as well. This allows collecting health data for each cohort according to their risk-factor status (longitudinal) or the health status of the population by age, sex, and risk-factor status by each year of the simulation (cross-sectional).

References

1. Boshuizen HC et al. Efficient implementation of risk factor/chronic disease Markov models for use in health impact assessment. Demography (accepted for publication); 2011

2. Barendregt JJ, van Oortmarssen GJ, van Hout Ben A., van den Bosch JM, Bonneux L. Coping with multiple morbidity in a life table. Math Popul Stud 1998; 7(1):29–49.

3. van Imhoff E, Post W. Microsimulation Methods for Population Projection. Population: An English Selection 1998; 10(1):97–138. Available from: URL: http://links.jstor.org/sici?sici=1169-1018%281998%292%3A10%3A1%3C97%3AMMFPP%3E2.0.CO%3B2-D.

4. Karnon J. Alternative decision modelling techniques for the evaluation of health care technologies: Markov processes versus discrete event simulation. Health Econ 2003; 12(10):837–48. Available from: URL: http://dx.doi.org/10.1002/hec.770.

| - Disease data (for every included disease)   - Excess mortality   - Incidence   - Prevalence   - RR given risk-factor class   - RR from other diseases (optional)   - DALY weights - Population data   - Size   - Newborns (optional*)   - Overall mortality   - Overall DALY weights - Risk Factors   - Prevalence (categorical, continuous, or by duration)   - RR for death (optional*)   - Transition probabilities between risk-factor classes (net transitions and zero transitions available as default)   *omitting RR for death implies a slightly different model interpretation than presented in article |
| --- |

Fig. 6: Required Input Data (age- and sex-specific)

1. Lhachimi SK, Nusselder WJ, Smit HA, van Baal P, Baili P, Bennett K, Fernández E, Kulik MC, Lobstein T, Pomerleau J *et al*: **DYNAMO-HIA-A Dynamic Modeling Tool for Generic Health Impact Assessments**. *PloS one* 2012, **7**(5):e33317. [↑](#footnote-ref-1)
